# Impact of the SARS-CoV-2 Spike Protein on the Innate Immune System: A Review

**DOI:** 10.7759/cureus.57008

**Published:** 2024-03-26

**Authors:** Annelise Bocquet-Garçon

**Affiliations:** 1 Immunology, Independent Researcher, Béthune, FRA

**Keywords:** type-1 interferons, cytokine storm, public awareness about vaccination, spike protein and covid-19, innate immune system

## Abstract

The Spike protein enables the severe acute respiratory syndrome coronavirus 2 (SARS-CoV-2) infection by binding to multiple receptors, including the angiotensin-converting enzyme 2 (ACE2). Scientific studies also indicate that Spike is involved in severe forms of coronavirus disease 2019 (COVID-19), "long-haul COVID diseases" - also known as "long COVID syndromes" or "post-acute sequelae of SARS-CoV-2 infection" (PACS) - or, recently, in adverse reactions to lipid nanoparticle-messenger ribonucleic acid (mRNA) vaccines or other anti-COVID19 products. Numerous mutations, notably within the subunit 1 of Spike (S1), prevent neutralization by antibodies, but more generally, the virus has developed numerous strategies to avoid immune system surveillance, especially type-I interferons (IFN-I). Meanwhile, a “hyperinflammatory” state, named “cytokine storm,” sets in. However, what role does the Spike protein play in the immune escape mechanisms? Can its inflammatory activities affect IFN-I? Does Spike block IFN-I or hijack them for the virus benefits? What are the other potential consequences? This article was written to provide an up-to-date and more general overview of the impact of the Spike protein on the innate immune system and its effectors at the molecular level.

## Introduction and background

Since the emergence of severe acute respiratory syndrome coronavirus 2 (SARS-CoV-2), many scientists have studied its structure, pathophysiological mechanisms, and evolution. One of the major viral proteins that have attracted the attention of the scientific community is the Spike protein [1,2].

Briefly, Spike is a homotrimeric structure, with each monomer being a protein composed of 1,273 amino acids with several functional domains and several proteolytic cleavage sites, including one furin cleavage site and two cathepsin L (CSTL) cleavage sites [1,3,4]. It is commonly accepted that subunit 1 of Spike (S1), comprising the N-terminal domain (NTD) and receptor-binding domain (RBD), is responsible for binding between the virus and its many cellular receptors or co-receptors [5]. The subunit 2 of Spike (S2), via a particular domain that is highly conserved among coronaviruses, is responsible for the fusion between the target cell and SARS-CoV-2 [1,2]. Spike is also a highly glycosylated protein, which may prevent some antibody/antigen interactions [6], and it mutates readily, particularly at the S1 subunit [7]. These mutations are thought to be responsible not only for the immune escape mechanisms of SARS-CoV-2 variants but also for their increased infectivity [8]. Recently, prion-like domains (PrDs) have been identified on the Spike protein, particularly in the RBD. It appears that mutations in these domains have increased the affinity of Spike for its major receptor, angiotensin-converting enzyme 2 (ACE2), particularly for the Delta variant [9]. Mutations in the NTD seem to increase the affinity of the Spike protein for gangliosides, lipid raft components of the plasma membrane, allowing SARS-CoV-2 to infect cells more rapidly [10].

Interestingly, many proteases appear to exert their proteolytic activities on the Spike protein. For example, via bioinformatics tools capable of predicting and prioritizing proteolytic cleavage sites on substrates, Bollavaram's team demonstrated that most cathepsins (CTSs) can potentially hydrolyze the Spike protein [11]. In the literature, it is possible to find other proteases, such as neutrophil elastase, plasmin, or trypsin, that can cleave Spike [12-14]. However, the most studied proteases are of course furin and transmembrane protease serine 2 (TMPRSS2). Of note, TMPRSS2 belongs to the type-2 transmembrane serine protease (TTSP) family, which has several members, including TMPRSS4, TMPRSS11d, and TMPRSS13, appearing as well to exhibit proteolytic activity on the Spike protein [15,16].

The last two proteases mentioned, i.e., TMPRSS11d and 13, even seem to facilitate the replication of SARS-CoV-1 and SARS-CoV-2 in vitro [16]. Nevertheless, recent studies on the Spike protein of the omicron branch variants demonstrate a reduced capacity for proteolysis by furin and TMPRSS2 [10]. Is it one of the reasons why these SARS-CoV-2 variants are less pathogenic? Probably, and this point will be discussed in this article.

The infection mediated by SARS-CoV-2 induces a pathology called "coronavirus disease 2019" (COVID-19) that results in a wide range of clinical manifestations, from asymptomatic cases to severe and life-threatening forms [17,18]. In general, patients with COVID-19 have respiratory disorders that can progress to acute respiratory distress syndrome (ARDS), as well as disorders in other organs, such as the liver or gastrointestinal tract, heart, central nervous system (CNS), and kidneys [18]. The sheer number of Spike receptors and co-receptors allowing viral entry, or potentially involved in this infectious disease, may account for the diversity of clinical presentations [5,19].

In addition, the level of disease severity could be associated with two mechanisms [20]. The first of these mechanisms, hyperinflammation (also called cytokine storm), is an overactivation of the innate immune system in conjunction with pyroptosis. This phenomenon can be defined as the death of virus-infected cells via inflammasomes and the production of proinflammatory cytokines, such as interleukin-1β (IL-1β) and interleukin-18 (IL-18), with the release of damage-associated molecular patterns (DAMPs), which alert macrophages and other innate immunity effectors, such as natural killers (NKs) [21,22]. This process represents the initiation of the normal antiviral immune response. However, the immune escape mechanisms, especially those mediated by the Spike protein, or molecular interactions between Spike and its receptors, such as ACE2, can disrupt the control of the immune system, as will be discussed below [23-25]. All these processes can then become problematic, leading to lethal multisystem failure [25].

Moreover, in the context of SARS-CoV-2-mediated infection, interferons (IFNs) appear to play a crucial role in combating the virus. Indeed, it was demonstrated that people with deficiencies in toll-like receptors (TLRs), notably toll-like receptor-3 (TLR3) and toll-like receptor-7 (TLR7), are more susceptible to the virus. IFN production is lower in these individuals, and they are therefore more likely to develop severe forms of COVID-19 [26,27]. Moreover, one of the mechanisms by which the virus escapes immune surveillance is by partially inhibiting type-I IFN (IFN-I) production [28,29]. Thus, the question is "does the Spike protein contribute to the IFN-I response escape?"

The second mechanism, which may be related to the hyperinflammation process, is immune cell depletion [20,25]. The virus can directly infect immune cells, leading to their lysis, but certain cytokines, type-II IFN (also called IFN-γ)) and tumor necrosis factor-α (TNF-α), can induce another phenomenon: PANoptosis.

PANoptosis can be defined as the coactivation of the pyroptosis, necroptosis, and apoptosis pathways, resulting in the establishment of a molecular complex, the PANoptosome. According to Karki and his team [30], this phenomenon may not only explain the tissue damages observed in severe cases of COVID-19 but also participate in lymphopenia [25,30]. Can PANoptosis be linked to the Spike protein effects? As described in this article, the Spike protein can induce the production of these two cytokines, IFN-γ and TNF-α, and so be related to PANoptosis.

Several other mechanisms can also explain the lymphopenia observed in severe cases of COVID-19. The Fas death receptor (Fas)/Fas Ligand (FasL) system (Fas/FasL system) is one of them. It consists of the expression of Fas, also called cluster of differentiation 95 (CD95), on the cell membrane of activated T cells. When stimulated by FasL, CD95 leads to the destruction of lymphocytes. Although FasL production is highly regulated due to its cytotoxicity, it appears that its plasma levels increase significantly with the severity of this infectious disease in correlation with the C-X-C motif chemokine ligand 10 (CXCL10) production [31]. An important observation must be highlighted: an increased production of IFN-γ and CXCL10 was detected following injections of modified messenger ribonucleic acid (mRNA) lipid nanoparticles [32,33]. Today, few studies allow us to understand how these cytokines are induced after the administration of these new vaccines [32,34]. Thus, can the Spike produced in the body following these anti-COVID19 injections be the origin of IFN-γ and CXCL10 synthesis? More broadly, what are the implications of Spike in the mechanisms inducing lymphopenia?

Finally, the Spike protein is likely to be involved in long COVID syndromes. Indeed, some results show the persistence of Spike protein over several months in the blood following infection, and for some patients, the plasma levels of this glycoprotein appear to coincide with COVID-19 vaccine administration. However, the authors do not specify which type of vaccine was used [35]. An important question that can be asked here is, how to explain the persistence of the circulating Spike protein over time? Normally, the effectors of the immune system should eliminate it. Why is this not the case?

In response to the pandemic, vaccination campaigns were conducted worldwide using a novel mRNA technology encoding the Spike protein, pharmaceuticals marketed by Pfizer and Moderna [36-38]. Note that the modified mRNA from these anti-COVID-19 injections, encoding the Spike protein, has two substitution codons leading to the placement of 2 prolines at positions 986 and 987. The purpose is to block the Spike protein produced in the prefusion state [39]. However, studies tend to demonstrate that this modified Spike, also called "Spike 2 prolines" (S2P) in some publications, can interact with ACE2 and can be cleaved by the furin protease [40,41]. Moreover, compared to the so-called "viral" Spike protein, there are few data regarding the structure, conformation, or glycosylation state of the so-called "vaccine" Spike, the one produced by the body's cells following injections of modified mRNA lipid nanoparticles. These data are necessary to establish the immunogenicity, antigenicity, and efficacy of these new vaccines since the strategy is ultimately to produce antibodies directed against Spike [42,43].

It is commonly admitted that vaccines containing modified mRNA encoding the SARS-CoV-2 Spike protein induce a high production of antibodies capable of neutralizing the virus Spike in weeks following their administration [44,45]. Furthermore, the experimental results show that anti-COVID19 injections induce high-affinity antibodies against the S1 subunit of the SARS-CoV-2 Spike protein but are less effective against the S2 subunit, consistent with the proline modifications mentioned above [45]. To our knowledge, antigen/antibody interactions are due to the recognition of linear or conformational antigenic epitopes via antibody paratopes [46]. Thus, it is possible to deduce that these two proteins, the viral Spike and vaccine Spike, have sufficiently strong structural and conformational homologies to justify the neutralizing abilities of the antibodies produced following the administration of these newly modified mRNA vaccines. Consequently, it is possible to talk about the Spike protein in a general term, whatever its origin.

Three remarks can then be made. First, S2 appears to possess highly conserved epitopes among coronaviruses, particularly at the level of the fusion peptide. Moreover, antibodies directed against this part of the Spike are broad spectrum, i.e., they neutralize a wide variety of *Orthocoronavirinae* viruses [47]. Thus, would it be interesting to consider a vaccine strategy against S2? Second, S1 seems to mutate the most [7,48], explaining the decrease of the immune protection, both cellular and humoral, conferred either by infection or by anti-COVID19 injections, against SARS-CoV-2 variants - particularly for the Omicron sublineage - as well as the observed changes in the virulence and pathogenicity of these strains [8,49]. Third, Spike shares similar sequences with human molecules, which may induce autoimmune processes or disrupt molecular interactions as it has been suggested for the Notch receptor [50,51]. Hence, what other repercussions can be envisaged at the pathophysiological and molecular levels, particularly on the functioning of immune cells, due to the presence of this Spike protein?

At this stage, it is important to draw attention to the second point mentioned above. If mutations in S1 allow the virus to evade immune protection, this may partly explain the infectious episodes observed in the early months of 2021 in subjects with a complete vaccination scheme [52]. Indeed, other explanations are possible for these breakthrough infections, such as antibody-dependent enhancement (ADE) or mechanisms inducing depletion of cellular effectors of the immune system, which were mentioned in a previous paragraph [53,54]. This brings us back to the same questions: What is the role of Spike in the mechanisms leading to lymphopenia? Is the current vaccine strategy the most relevant one? What are the impacts of Spike protein, since it is possible to talk about it in general, on the immune system?

To conclude this introductory section, the study of the potential impacts of the Spike protein on the immune system must address the following points: How is Spike involved in the development of cytokine storms? How does Spike escape the immune system, especially IFN-I? Does dysregulation of IFN-I contribute to the cytokine storm? And if so, to what extent? Does it participate in lymphopenia or induce an immune tolerance that explains the persistence of Spike in the body?

The objective of this article is to bring some answers to these questions through a review of the scientific literature, research carried out on the specialized sites PubMed or National Center for Biotechnology Information (NCBI).

## Review

The Spike protein triggers cytokine storms

At the beginning of a viral infection, host cells can detect the presence of pathogens by the pattern recognition receptors (PRRs). These receptors are widely distributed onto and into cells, and their activation depends on the pathogen-associated molecular patterns (PAMPs) present in the virus [55]. Highly conserved among pathogens categories, PAMPs can be described as specific molecular structures made of lipids, nucleic acids, and proteins. Belonging to innate immunity, PRRs can be defined as sensors of PAMPs by binding to their respective ligands. According to the current state of knowledge, most of these sensors are classified into five types: toll-like receptors (TLRs), nucleotide oligomerization domain receptors (NLRs), retinoic acid-inducible gene-I receptors (RLRs), C-type lectin receptors (CLRs), and absent-in-melanoma-2 (AIM2) receptors (ALR) [56]. Generally, the activation of PRRs leads to the translocation of the nuclear factor-kappa B (NF-κB) into the nucleus and the activation of the mitogen-activated protein kinase (MAPK) pathway, which induce the production of proinflammatory cytokines with IFNs and their release. The development of inflammasomes, which leads to pyroptosis, i.e., the death of infected cells, induces the release of IL-1β, IL-18, and damage-associated molecular patterns (DAMPs) [21,25,56]. The purpose of all these molecules, i.e., cytokines and IFNs, is to start an efficient antiviral immunity via the recruitment, the activation of immune cells, and the orientation of the adaptative immune response [57].

In the case of SARS-CoV-2, studies to identify the PRRs involved in the activation of the innate immune response are still ongoing. It appears that TLRs, RLRs, NLRs, and cyclic GMP-AMP synthase (cGAS) - stimulator of IFN genes (STING) pathway (cGAS-STING pathway) - a mechanism induced when some nucleic acids are present in the cell cytoplasm, are activated, triggering the production of proinflammatory cytokines and IFNs in the early stages of infection [58,59]. However, SARS-CoV-2 has a molecular arsenal capable of limiting PRR signaling, thus limiting the production of inflammatory cytokines and IFNs, allowing the virus to escape the immune system rapidly. This explains, at least in part, the spread of the virus in the body in the late stages of the disease and then the exaggerated innate immune response, called a cytokine storm [58].

Is There a Molecular Mechanism That Induces an Amplification Loop of ACE2 and TLR4 Expression?

The main cytokines associated with cytokine storms during SARS-CoV-2 infection are interleukins (IL-2, IL-4, IL-6, IL-7, IL-10 plus IL-8 and IL-17), IFN-γ, tumor necrosis factor (TNF-α), granulocyte-colony-stimulating factors (G-CSF), and chemokines [60-62]. Even if two other viral proteins, i.e., envelope (E) and nucleocapsid (N) - may interact with TLR2 [63-65], the Spike protein can also be involved in its activation [66]. The experiments carried on macrophages and epithelial cell cultures showed that these cells sense both recombinant S1 and S2 subunits or the trimeric Spike protein via TLR2, activating the NF-κB pathway and, to a lower extent, the MAPK pathway, to produce these kinds of proinflammatory cytokines and chemokines. Intriguingly, no production of type I or type II IFNs was detected after human leukemia monocytic cells-derived macrophages (THP1-derived macrophages) stimulation, and the TLR4 seemed to not be involved in the Spike-induced inflammation [66].

Notwithstanding, if the molecular basis is still unknown, a direct interaction between the SARS-CoV-2 Spike protein and TLR4 was demonstrated by a surface plasmon resonance assay [67]. TLR4 can also be implicated in the inflammatory process mediated by Spike, including in the central nervous system (CNS), and these data are corroborated by different publications [68-70]. According to Aboudounya and Heads [70], the interaction between Spike and TLR4 leads first to the activation of the canonical pathway, initiated by the myeloid differentiation primary response 88 (MyD88) and resulting in the translocation of the NF-κB factor, and second the activation of the MAPK pathway via the transforming growth factor-β (TGF-β)-activated kinase 1 (TAK1), to produce proinflammatory cytokines and chemokines. The activation of the non-canonical pathway, via the toll/interleukin-1 receptor (TIR)-domain-containing adaptor-inducing beta interferon (TRIF) and TRIF-related adaptor molecule (TRAM) system (TRIF/TRAM system), is also possible. This molecular cascade induces the production of anti-inflammatory cytokines, such as IL-10, and, above all, the production of IFN-I [70].

Thus, both Spike/TLR2 or Spike/TLR4 interactions can be, ipso facto, involved in an inflammatory process via the activation of the NF-κB and MAPK pathways.

Then, in an autocrine amplification loop, by binding to interferon alpha-beta receptors (IFNARs), IFN-I induces the expression of interferon-stimulated genes (ISGs) [70]. Although controversial, a direct relationship between ACE2 expression and IFN-I pathways probably exists, with ISGs increasing the expression of ACE2, the main Spike protein receptor [71-74]. The differences between the results reported in these publications can be explained by the experimental protocols used, in vivo experiments being different from those in vitro, the cell lines used, the kind of ISGs focused, and, above all, the pathophysiological context.

Of interest, the viral mRNA can be sensed by RLRs, such as the melanoma differentiation-associated protein 5 (MDA5), which interacts with the mitochondrial antiviral signaling protein (MAVS) [25]. This mechanism triggers an IFN response, and MAVS was identified as an ISG [75-77].

Using human cell models, Yan and his colleagues [77] found that the stimulation of RLRs with viral RNA mimic poly(I:C) or viral DNA mimic poly(dA:dT) increases the level of both ACE2 mRNA and protein, 13 ISGs promote its expression (especially ISG95), and finally, four of them (ISG10, ISG52, ISG71, and ISG95) can significantly enhance the NF-κB level. All these data suggest that ACE2 behaves like an ISG and its level correlates positively with NF-κB activities [77]. Thus, it is possible that the recognition of the Spike protein by TLR4 participates in the IFN-I response, which, in turn, improves ACE2 expression.

Another point to highlight is that in a recent publication dated September 2022, the researchers stimulated various TLRs localized on macrophages and peripheral immune cells (PBMCs), including TLR4 with lipopolysaccharide (LPS), resulting in the translocation of ACE2 and TMPRSS2 to their surface. While global ACE2 synthesis in these cells is not promoted, it may render them sensitive to the Spike protein once their TLR4 is stimulated [78].

Remarkably, the interaction between Spike and ACE2 may contribute substantially to an immune overactivation. Indeed, the binding between these two elements may disrupt the renin-angiotensin-aldosterone system (RAAS) by making the ACE2 receptor unavailable for angiotensin II degradation. This can lead to an overactivation of the angiotensin II type 1 receptor (AT1R) and the production of proinflammatory cytokines, such as IL-6, TNF-α, and IFN-γ [23,24]. As it was described in the introduction, TNF-α and IFN-γ synergize to induce PANoptosis. Thus, Spike can be directly involved in this destructive phenomenon via its interaction with ACE2 itself, resulting in an angiotensin II accumulation that leads to an amplification of the TNF-α and IFN-γ secretion. IL-6 will induce another phenomenon, detailed below in this article.

In addition, binding of the Spike protein to ACE2 leads to its internalization, resulting in a decrease in ACE2 enzymatic activity. The result is not only an increase in angiotensin II but also an accumulation of bradykinin. In fact, ACE2 also controls the kinin system by eliminating bradykinin, which is also implicated in inflammatory mechanisms. As a result, an imbalance occurs in the kinin system, leading to a "kinin storm" [79,80].

Interestingly, angiotensin II also exerts direct activity on immune cells by recruiting them to the inflammatory site and activating them. It also stimulates the expression of a particular receptor of great interest in the context of this article: TLR4 [23,81]. This is already a true amplification loop of a Spike-induced proinflammatory response with Spike/TLR4 interaction-IFN I activation-ISG expression and ACE2 upregulation/Spike interaction-angiotensin II accumulation-TLR4 expression enhancement.

What Role Do LPS and the Amyloid Properties of the Spike Protein Play in Inflammation?

It seems that the Spike protein, or the S1 and S2 subunits independently, can interact with bacterial LPS that is recognized by TLR4 [82-84]. LPS is a component of the outer membrane of Gram-negative bacteria, such as *Porphyromonas* *gingivalis*, *Escherichia coli,* or *Pseudomonas aeruginosa*, all common pathogenic or commensal bacteria. As Petruk and colleagues wrote [82]: “All these observations on links between LPS levels and several diseases and conditions, along with the risk for developing severe COVID-19, imply that measurement of endotoxin levels in COVID-19 patients could have significant diagnostic implications and be of relevance for patient management and treatment decisions*.*” This sentence is truer in light of the data exposed above.

Another point to note: LPS aggregates with the Spike protein, leading to the formation of amyloid structures [83]. Interestingly, the Spike protein, cleaved by neutrophil elastase, can form amyloid fibers, and one Spike sequence can change its conformation from an α-helix structure to a β-sheet under certain conditions and agglomerate to form a hydrogel film [12,85]. In addition, the S1 subunit can induce neuroinflammation independently of SARS-CoV-2 infection [86]. This neuroinflammation can lead to an overexpression of PrPc, the normal form of the prion protein, and an activation of the amyloid precursor protein (APP) [87]. Moreover, the S2 subunit has been shown to interact with γ-secretase, an enzyme involved in the processes of amyloid beta-42 (Aβ42) formation from APP, increasing the production of this type of fibril [88]. Tied together, all these data indicate that Spike may potentiate or even accelerate underlying neurodegenerative diseases, contributing to their progression [89]. Not only can Spike itself be amyloid, but it can also interact with other amyloid fibers; notably the S1 subunit can bind Aβ42 [90,91]. This S1/Aβ42 interaction appears to increase the production of IL-6, one of the most abundant proinflammatory cytokines found in severe forms of COVID-19, via activation of ACE2. In fact, binding with Aβ42 does not impede the S1 subunit from binding with its receptor [91]. It is also known that TLR2 and TLR4 are involved in neurodegenerative diseases, such as Alzheimer's, even if their roles in this pathology remain under investigation [92,93]. For example, some experiments carried out on a mouse model showed that TLR2 deficiency aggravates the loss of neurons and white matter damage, even though TLR2 is a therapeutic target because of the inflammatory processes [92]. TLR4 can also sense amyloid beta (Aβ) aggregates and is implicated in the inflammatory response that leads to the progression of Alzheimer's disease [93].

Thus, by binding to this type of fibril, does the S1 subunit of Spike (or the Spike full length) “overactivate” both TLR2 and TLR4? And does LPS intervene in both ACE2 and TLR4 activation when combined with S1/Aβ42 complex?

And does the so-called “vaccine” Spike protein trigger the same effects?

The structure of the “vaccine” Spike protein is very similar to that of the "viral" Spike, as explained in the introductory section. It can also bind to ACE2 [39,44] and has a higher affinity for LPS than the viral Spike due to the two proline modifications [84]. In addition, since it retains the furin cleavage site, it can be dissociated into two subunits, S1 and S2 [40,41]. It still needs to be demonstrated whether the S2 subunit, with its two proline modifications, can also interact with the γ-secretase. However, it is possible that this so-called “vaccine” Spike can trigger the same phenomena as those described above.

Moreover, MDA5 is implicated in the detection of the modified mRNA present in the lipid nanoparticles [34,94]. Using knockout experiments in a mouse model, Li and colleagues demonstrated that no other PRR than MDA5 is involved in CD8+ T cells' response to immunization with BNT162b2 and correlated with IFN-I and IFN-II production [34]. They also found an upregulation of ISG15, the one playing a role in the production of a truncated isoform of ACE2 (dACE2) of unknown biological function [34,72]. Further investigations are needed to understand how ACE2 responds as an ISG and why a truncated isoform is probably produced.

ISG15 expression can be mediated by MDA5, as this type of PRR can induce IFN production. ISG15 is even more important because it promotes MDA5 activity through a mechanism known as ISGylation [95]. Briefly, ISG15 is a small 17 kDa protein containing two ubiquitin-like domains. It is rapidly transformed into its mature 15 kDa form, exposing a particular C-terminal motif. ISG15 is then covalently bound to its target proteins via enzymes, a process called ISGylation [96]. The work carried out by Liu's team [95] shows that MDA5 is ISG15-conjugated at the lysine (K) residues in position 23 and 43 of its caspase activation and recruitment domains (CARDs), and this ISGylation is necessary for the activation and activity of this PRR, enabling an IFNβ (a type-I IFN) production.

According to Nicholas A. Mathieu’s paper, ISG15 is induced by IFN-I with all its conjugating enzymes [97]. One of these enzymes, homologous to E6-associated protein carboxyl terminus (HECT) and regulator of chromosome condensation 1 (RCC1)-containing protein 5 (HERC5), appears to play an important role in protein ISGylation, particularly in the conjugation of viral proteins. Indeed, HERC5 ISGylates nascent proteins during translation, and the current predominant theory is that HERC5 targets newly translated viral proteins, their ISGylation serving to disrupt their functions [97]. This raises the question of what impact ISG15 expression in cells, which have incorporated some modified mRNA contained in nanoparticles, might have on Spike protein production and, consequently, on anti-Spike antibody production in this vaccine context.

In addition, ISG15 displays other biological functions. Indeed, ISG15 is a key immunomodulator, which, in synergy with the ubiquitin-specific protease-18 (USP18) - the only enzyme able to deISGylate proteins, i.e., to remove ISG15 from its target proteins - is involved in the negative control of the IFN-I response [98]. Both can be linked to the interferon regulatory factor-9 (IRF-9) activation and regulation [99]. Moreover, ISG15 and its entire conjugating system can be induced by tumor protein p53 (p53) under DNA damage circumstances. In return, p53 is ISGylated to increase the binding of p53 to the promoter region of its target genes and its own gene, creating an amplification loop [100]. Secreted from monocytes, lymphocytes, or granulocytes, ISG15 acts as a cytokine that stimulates synthesis and secretion of IFN-γ through its interaction with the leukocyte function-associated antigen-1 (LFA-1), a molecule expressed by immune cells including NK and T cells. More precisely, ISG15 cooperates with IL-12 to induce IFN-γ and IL-10 production by NK [101].

Interestingly, IL-12, IL-10, and IFN-γ were detected in the muscle alone or in the serum or both in both the muscle and serum of mice injected with modified mRNA nanoparticles accompanied by TNF-α and other cytokines [102]. Moreover, the main cytokines detected in the serum of people injected with modified mRNA encapsulated in lipid nanoparticles, specifically BNT162b2 (Pfizer), were IL-6, TNF-α, IFN-γ, and CXCL10 [32,33,103]. It also appears that certain monocyte populations are epigenetically conditioned to respond more intensely to vaccine booster doses, with increased production of IFN-γ and CXCL10 [32,34]. Similarly, TNF-α levels also increase significantly after boosters, concomitant with IFN-γ and anti-spike antibody productions [34,103]. It is therefore possible that a PANoptosis phenomenon may occur, particularly following repeated injections of lipid nanoparticles and Spike protein synthesis. This injection-induced multisystem inflammation syndrome (MIS) is just as dangerous as that triggered by the virus, as reported in the scientific literature, in both adults and children [104-108].

Another pathology of concern may be linked to exposure to Spike protein and/or anti-COVID19 injections associated with CXCL10 production: myocarditis [109-111]. Studies have shown an immune infiltration in the endomyocardium composed mainly of T lymphocytes, sometimes associated with macrophages, and more rarely an infiltration of eosinophils [111], which is congruent with a CXCL10 synthesis [109,112].

The Spike protein interferes with the type-I IFN response

IFNs are classified into three groups: type-I IFNs (IFN-I), type-II IFNs (IFN-II), and type-III IFNs (IFN-III). IFN-I comprise a variety of genes, including 13 IFNα, IFNβ, IFNω, IFNε, and IFNκ. IFN-II, also known as IFN-γ, is structurally distinct from IFN-I, and there is only one known representative of this class to date. The IFN-III family, also known as IFN lambda, represents members of the IL-10 superfamily and includes the IFNλ1 (IL-29), IFNλ2 (IL-28A), IFNλ3 (IL-28B), and IFNλ4 genes [76].

In this article, the focus is on IFN-I because they are involved in antiviral processes, from the generation of an antiviral state to the orchestration of innate and adaptive immune responses. Secreted type-I IFNs bind to a receptor, a heterodimeric IFN-I receptor (IFNAR), and trigger the Janus kinase (JAK)/signal transducers and activators of transcription (STAT) signaling pathway (JAK/STAT signaling pathway), leading to the expression of antiviral genes. In this way, certain cellular translation processes are blocked, making it difficult for the virus to replicate within the cell. Not only do they trigger a state of cellular resistance to the virus, but they also alert neighboring uncontaminated cells and induce cytokine production to warn the immune system. In fact, IFN-I also plays an important role in initiating and enhancing antigen presentation, as well as acting directly and indirectly on T and B cells to trigger adaptive immune responses [76,113].

To schematically summarize the publication by Lukhele and his team [76], the activation of the PRRs, including MDA5 discussed previously, induces the phosphorylation, activation, and dimerization of the IRF-3, as well as the release of the NF-κB. Then, IRF-3 and NF-κB move into the nucleus, inducing the transcription of IFN-I (especially IFNβ) and proinflammatory cytokines. After their release in the cell environment, IFN-I acts in an autocrine and a paracrine way via their receptor (IFNAR), creating a positive feedback loop that triggers the synthesis of various members of the IFN-I family in a second burst of IFN-I production. In this context, IRF-9 is involved in the induction of IFN-stimulated gene expression (ISG) via a molecular complex called “interferon-stimulated gene factor 3” or ISGF3 [76]. Our focus will therefore be on two elements: IRF-3 and IRF-9.

Does the Spike Protein Specifically Target IRF-3?

The recombinant Spike protein seems able to suppress IFN-I when incubated with primary macaque bronchoalveolar lavage (BAL) cells [114]. Of note, Sui and colleagues [114] demonstrated that IFN-I inhibition induces a decrease in ACE2 expression, which is consistent with the analysis carried out in the first part of this review. Other analyses also indicated that Spike specifically suppressed IRF-3 expression, while levels of the transcription factors NF-κB and STAT-1 remained intact. Thus, Spike may block IFN-I, thus partially disrupting innate immunity, but maintaining the synthesis of proinflammatory cytokines [115].

Interestingly, Freitas and his colleagues were unable to determine whether the full-length Spike protein or the S2 subunit is capable of inducing IRF-3 degradation in the proteasome [115]. The results tend to favor the full Spike over S2, but this remains to be investigated. Most interestingly, proteasome inhibition not only restored IRF-3 expression in the presence of Spike but also increased Spike expression and minimized its proteolytic cleavage [115]. The use of proteasome inhibitors has also been suggested to block SARS-CoV-2 replication and limit the cytokine storm [116,117]. It is therefore possible that Spike undergoes host cell-mediated degradation in the proteasome, leading to its activation. This hypothesis is reinforced by the trypsin-like activity of the proteasome. Indeed, trypsin cleavage of Spike leads to virus fusion with target cells in vitro [14,118].

Thus, the Spike protein may be activated in the proteasome, while inhibiting the IFN-I response.

Numerous publications report the formation of syncytium due to Spike's fusogenic properties [119-123]. Syncytia are structures formed by the "fusion" of several cells, and these are called "cell-in-cell structures" (CIC), literally "structures of cells within cells." Of note, these structures can integrate lymphocytes, internalizing and destroying them, thus contributing to the development of lymphopenia. Zhang and colleagues also mentioned that the formation of heterotypic CIC structures constitutes a mechanism of immune evasion in tumors by consuming functional immune cells, such as T lymphocytes or NK [121].

Intriguingly, one study shows that the formation of cell syncytia reactivates IRF-3, and therefore IFN-I, via the cGAS-STING pathway. Indeed, Liu and his team demonstrated that as the Spike protein is produced on the cell surface, cell fusions occur, leading to the rupture of nuclear membranes and the formation of micronuclei [124]. These micronuclei are detected by cGAS, which activates STING, triggering IRF-3 phosphorylation and IFN-I production. This is followed by the induction of interferon-induced transmembrane proteins (IFITMs), a family of restriction factors, blocking Spike-mediated cell fusion [119,124]. However, the serine protease TMPRSS2 enhances syncytia formation by accelerating the fusion process and counteracts the antiviral effect of IFITMs [119].

In addition, a study shows that the Spike protein interacts with IFITMs, contributing to SARS-CoV-2 infection [125]. It is therefore reasonable to think that the innate response via type-I IFNs would be ineffective in countering the virus and the cell fusion phenomenon, unless another therapeutic strategy is used to modulate this immune response, as suggested in a preprint by Hoang's team [126].

According to Lista and Winstone, resistance to type-I IFNs and IFITMs depends on variants and mutations affecting the Spike protein [127]. For example, the alpha variant appears to be more resistant to IFN-I and the infection seems to be enhanced by IFITM-3 due to the P681H mutation on Spike, near the furin cleavage site. This mutation also affects the tropism of the virus, enabling it to avoid the endosomal compartment where IFITM-2 is located [127]. Interestingly, the Spike of the Omicron variants presents the same P681H mutation as the Spike of the alpha variant. However, studies show that all mutations affecting the Spike protein of the Omicron lineage impact the development of IFITM resistance, being sensitive to IFITM-1, IFITM-2, and IFITM-3 and to other restriction factors belonging to the ISGs, like guanylate-binding proteins 2 and 5 [127,128]. Thus, normally, infections mediated by Omicron viruses are prone to be attenuated by an IFN-I response. Curiously, this is not the case, and variants of the Omicron lineage show, on the contrary, increased resistance to type-I IFNs [29,129].

Of course, IFN-I escape may be due to mutations affecting other SARS-CoV-2 proteins, such as the nucleoprotein N or the non-structural proteins (NSPs), or through other mechanisms [29,130]. However, by analyzing the above data, it is possible to ask whether mutations in the Spike protein disrupt or enhance its interaction with IRF-3. This question needs to be investigated. Also, one may ask how can Spike of Omicron variants contribute to their IFN-I escape.

In a publication, researchers demonstrate that isolated Spike, i.e., without the presence of endotoxin, specifically activates TLR4 but without inducing TIR-domain-containing adaptor-inducing beta interferon (TRIF), an adaptor molecule used by certain intracellular-signaling TLRs, notably TLR3 and TLR4 [131,132]. Not activating TRIF means not inducing IFNβ expression via IRF-3 [133]. This phenomenon explains why TLR3 deficiency is an aggravating factor in SARS-CoV-2 infection, as Spike/TLR4 interaction alone cannot induce IFN-I production [26,134].

At the same time, signal transduction initiated by TLR4 via MyD88 is maintained, leading to the translocation of NF-κB and thus proinflammatory cytokine production [132]. These data are important because TLR4 can be located on both the cell surface and the endosomal compartment, with TRIF activation attributed to the endosome [131,133]. This would mean that the Spike protein is detected by TLR4 at the cell surface and not in the endosome, as proteolytic cleavage of the Spike protein causes it to lose its interaction with this PRR. The Spike/TLR4 interaction would therefore be preferentially localized to the S1 subunit. This hypothesis is confirmed by different studies, but the mechanisms underlying TLR4 activation by the Spike protein are poorly understood and need further investigations [135].

Of note, the binding affinity to TLR4 for the Omicron Spike protein seems less than the “wild-type” Spike protein, and so, the induction of the inflammatory process seems less dangerous [132]. The Spike protein of Omicron variants also has less affinity for LPS, and that contributes to the lower pathogenicity of this viral line [84]. However, the amyloidogenic properties of the Spike protein of Omicron variants BA.1 and BA.2 are enhanced by mutations affecting the RBD domain. This results in a stabilization of the Spike protein in the "up" conformation (and hence greater affinity for its ACE2 receptor), enhanced thermal stability, and, potentially, more pronounced amyloid interactions, notably with heparin or fibrinogen, which may impact coagulation [136].

The amyloid peptides in the Spike protein of Omicron variants need further studies, particularly focused on the interactions with other fibrils, such as Aβ42, and their impacts on the immune system, especially on the TLR4 activation.

Is the Spike Protein of Omicron Variants Less Dangerous?

The Spike protein in Omicron strains may be less sensitive to the action of transmembrane serine proteases, notably TMPRSS2, TMPRSS11d, and TMPRSS13, due to mutations, and their fusogenic capacities therefore appear impaired [137-139]. Studies tend to show that Omicron variants prefer the endosomal entry route to that of the fusion at the plasma membrane [139,140], although the independence of their Spike protein from TTSPs may be questioned, particularly for BA.2.86 [141,142].

In fact, another molecular mechanism can be explored to investigate the Omicron strains infectious ways: the cleavage of membrane ACE2 via the membrane type 1 matrix metalloproteinase (MT1-MMP), also called matrix metalloproteinase 14 (MMP14), which releases this Spike’s receptor into a soluble form, solACE. This phenomenon can promote SARS-CoV-2 cell entry through receptor-mediated endocytosis, which is much more enhanced by the fusion of the Spike/solACE2 complex with AT1R or arginine vasopressin receptor 1B (AVPR1B) [143-145].

In the endosome, the Spike protein can be processed by cathepsins (CTSs), especially CTSL [139,146]. The experimental data demonstrated that the SARS-CoV-2 Spike protein presents many different cleavage sites, other than those of furin and TMPRSS2, and it is efficiently proteolyzed by CTSL with fusogenic activities clearly developed after CTSL cleavage. In addition, the H655Y substitution in the Omicron strains Spike protein consistently increased both cathepsin L and cathepsin B usage [4,139,146]. Finally, the N679K and P681R mutations appear to provide new cleavage sites for the Spike protein in Omicron variants, making it sensitive to other proteases such as cathepsin G produced by neutrophils [147,148].

Interestingly, Zhao and colleagues have concluded that SARS-CoV-2 can probably upregulate CTSL expression by an unknown mechanism [146]. In fact, CTSL expression is diminished by microRNA-200c (miR-200c), a small non-coding RNA, which is also implicated in the COVID-19 disease progression [149,150]. Analyses carried out on blood samples from critically ill patients showed a significant elevation of this microRNA (miR), miR-200c, in patients who died compared to those who recovered, confirming previous data on this subject [150-152]. The researchers suggested that miR-200c downregulates ACE2 expression, thereby increasing its depletion on the cell surface and hyperinflammation via the angiotensin II/AT1R axis in an infectious context [152].

However, miR-200c expression can be upregulated by LPS or lipoteichoic acid (LTA) through TLR4 and TLR2 activation, respectively, leading to the translocation of NF-κB and this microRNA synthesis [153]. miR-200c can therefore be linked to inflammatory processes. Curiously, in another study, miR-200c levels were downregulated in SARS-CoV-2-infected patients admitted to hospital, and this decrease in miR-200c is associated with an increase in the blood levels of IL-6. Discharged patients retained higher levels of IL-6 and lower levels of miR-200c than healthy individuals [154]. To interpret these data, it is necessary to contextualize the activity of the Spike protein, a context that may depend on several elements as explained in the discussion part.

Of interest, it was demonstrated that miR-200c downregulates contactin-1 (CNTN1) expression, impacting the RLR-mediated IFN-I signaling pathway. CNTN1 induces MAVS proteasomal degradation in synergy with ubiquitin-specific protease-25 (USP25), decreasing the expression of IFNβ and IFN-stimulated genes, notably ISG15. Xu and colleagues also suggested that CNTN1 can block the activation of TBK1 and IRF-3 [155]. CNTN1 plays different biological functions: At the plasma membrane level, this molecule is involved in cell adhesion and is highly expressed in the CNS [155].

Intriguingly, the Spike protein seems to interact with CNTN1 via its receptor binding domain (RBD), potentiating viral infection through ACE2. The study also showed that CNTN1 expression is significantly correlated with the viral load in COVID-19 patients [156]. However, the Spike/CNTN1 interaction has not been tested at the intracellular level. Therefore, by analyzing the data outlined above, one may wonder, what are the repercussions of this Spike/CNTN1 interaction in the immune response in connection with type-I IFNs?

Does the "Vaccine" Spike Protein Interact With IRF-3?

This question needs to be investigated because, after transfection of mRNA into cells, some of the Spike proteins produced are certainly processed in proteasomes, enabling the presentation of antigenic peptides on major histocompatibility complex I (MHC I). Injections of lipid nanoparticles containing modified mRNA, such as Pfizer's BNT162b2, further appear to induce a type-1 T helper cell (Th1) immune response rather than a type-2 T helper cell (Th2) response [157].

These data are consistent with one another and with stability studies of SARS-CoV-2 viral proteins [158] and antigen priming mechanisms on major histocompatibility complexes (MHCs) [159]. So, if some Spike proteins produced after transfection of modified mRNAs are processed in the proteasome, can they interact with IRF-3 leading to its degradation? This question is important to elucidate as IRF-3 amplifies and cooperates with IRF-7 during the second phase of the type-I IFN response [160,161], with IRF-7 presented as a key modulator involved in both pro- and anti-inflammatory processes [162].

Interestingly, another molecular complex is involved in the amplification loop of the type-I IFN response, which increased IRF-7 expression: the ISGF3, composed of IRF-9 and transcription signal transducers and activators 1 and 2, also known as STAT1 and STAT2 [160,161].

A Relation Between IRF-9, Spike, and miR-148a: Is It Another Proinflammatory Process?

Using a cell transfection technique with a plasmid encoding the SARS-CoV-2 Spike protein, Mishra and Banerjea found Spike being released in exosomes with microRNAs (miRNAs) [163], small non-coding RNAs, around 20 nucleotides long, deriving from genome expression and involved in its post-transcriptional regulation [164]. These authors especially identified miR-148a and miR-590 [163]. Once internalized by human microglia, the “brain-resident macrophages,” miR-590 targets directly IRF-9, whereas miR-148a downregulates the expression of the ubiquitin-specific peptidase 33 (USP33), thereby destabilizing IRF-9 levels in these immune cells. However, does this mean disrupting the ISGF3 formation and thus lowering IRF-7 expression? IRF-7 is involved in the transition from a proinflammatory M1-like phenotype to an anti-inflammatory M2-like phenotype of activated microglial cells. Indeed, a decrease in IRF-7 levels can block them in the M1-type phenotype, leading to neuroinflammation and lesions in the CNS [165].

By binding to IFN-stimulated response elements (ISREs) in the promoters of target genes, the ISGF3 complex plays an important role in the expression of several molecules, including ISG15, USP18, and p53 [99,166]. USP18 conjugated with ISG15 inhibits the type-I IFN signaling via its interaction with STAT2, modulating the IFN-α/β receptor 2 (IFNAR2) complex to avoid a hyperactive type-I IFN signaling, a phenomenon implicated in type-1 interferonopathies [99,167-169]. So, by targeting IRF-9 via miR-148a, does the viral Spike protein induce interferonopathies? It is a possibility.

Moreover, IRF-9 displays a particular relation with p53. To summarize, if IRF-9 can induce p53 expression via ISGF3, p53 can in turn induce IRF-9 expression [166]. In addition, IRF-9 suppresses gene expression of the silent mating-type information regulator two homolog 1 (SIRT1), which is responsible for p53 inhibition [170]. Thus, by targeting IRF-9 via miR-148a, can the viral Spike protein disrupt p53 expression and activities? This question is of major interest since p53 deficiency can aggravate inflammation, particularly in the lung [171], and the S2 subunit of the Spike protein can interact with p53 [172].

Various studies have shown that miR-148a is upregulated in patients with a severe form of COVID-19 [173,174]. Interestingly, de Gonzalo-Calvo and colleagues found a correlation between miR-148a dysregulation and neutrophils, platelets, C-reactive protein (CRP), and, most importantly, leukocyte count [173]. However, these parameters require further study.

MiR-148a is upregulated in M1-like macrophages and inhibits the signal regulatory protein α (SIRPα), a negative regulator of macrophage phagocytosis [175]. However, does the Spike protein induce miR-148a expression in macrophages? It was demonstrated that miR-148a expression is upregulated by Notch signaling, playing a role in the differentiation of monocytes into macrophages and in macrophage polarization toward an M1-like phenotype [176]. A model was presented in which Notch is involved in SARS-CoV-2 infection, enhancing viral entry into cells by promoting furin synthesis and downregulation of a disintegrin and metalloproteinase 17 (ADAM17), exacerbating inflammation in a Notch/IL-6 positive feedback loop and, finally, inhibiting lung regeneration [177].

Since the Spike protein can induce IL-6 production via the angiotensin II/AT1R axis, it can activate Notch. Notch activation is also known to depend on the γ-secretase complex [178]. Here, too, the interaction S2/γ-secretase raises questions [88,178]. Of interest, curcumin, retinoic acid, and other molecules could modulate the Notch signaling system [179].

Is There a Tolerogenic Effect via miR-148a Downregulation?

In another study, researchers found a slight decrease in miR-148a in nasopharyngeal tissue samples from people infected with SARS-CoV-2 [180]. The sampling site can explain this result, as mucous membranes are not necessarily representative of the systemic environment, especially in immunology [181].

However, downregulation of miR-148a expression can upregulate the human leukocyte antigen G (HLA-G), a key immunomodulator of immune responses [182]. HLA-G plays an important role in modulating innate and adaptive immune responses, in both healthy and pathological conditions, by inhibiting CD8+ T cells, NK, B cells, and dendritic cells (DCs) [182-184]. HLA-G can be expressed on the cell surface but can also be produced in soluble isoforms [183,185]. 

Different immune cells can express HLA-G on their surface, being characterized as HLA-G+ cell subpopulations [183]. Specifically, a certain subpopulation of DCs, called DC-10 because of its ability to produce an anti-inflammatory cytokine, IL-10, also expresses HLA-G and induces type 1 regulatory T cells (Tr1) [183]. While HLA-G expression is stimulated by a variety of factors, including proinflammatory cytokines, IL-10 appears to be an important inducer [183,186,187].

Interestingly, Seliger and colleagues explained in the introductory section that, in the early stage of SARS-CoV-2 infection, hosts produce some IL-10 leading to HLA-G expression [184]. Their experimental data showed an increase in lung-specific HLA-G expression in patients infected with SARS-CoV-2, a positive correlation between HLA-G expression levels and immune cell infiltration, a slight decrease in HLA-G expression correlated with nucleocapsid detection and localization, and a lower HLA-G expression in patients who died within seven days of infection. Of note, miR-148a seems to not be involved in HLA-G expression by SARS-CoV-2, but the results obtained by Seliger's team are unclear for the Spike protein and are part of a global infectious context [184].

In addition, it has been shown that the Spike protein or the S1 subunit alone can polarize macrophages or monocytes toward a proinflammatory phenotype (M1) with the production of TNF-α, IL-1β, IL-6, IL-8, and IL-10 [188,189]. This is not aberrant since, in myeloid cells, the expression of IL-10 is induced following the activation of PRRs, notably via the MAPK pathway [190]. So, via its TLR4 or TLR2 interactions, the Spike protein or the Spike/LPS complex can induce an IL-10 production by activating MAPK.

Spike can also interact with the cluster of differentiation 4 (CD4) allowing infection of CD4+ T lymphocytes and triggering IL-10 production [191]. This could lead to lymphopenia and extinction of the immune response, with the expression of potent immunomodulators, like IL-10 and HLA-G.

Do Modified mRNA Products Downregulate miR-148a?

After collecting blood samples from patients who had received two injections of BNT162b2, Miyashita and colleagues extracted extracellular vesicles that include exosomes, ectosomes, and apoptotic bodies and characterized their miRNA content [103]. Of interest, they also found a slight decrease in miR-148a. Intriguingly, they correlated this decrease in miR-148a with the level of antibodies produced following the administration of Pfizer's anti-COVID-19 product [103].

Two points can be formulated. The first one is, because of a miR-148a decrease, the immunomodulatory mechanisms described above may come into play. The second point is that, normally, levels of miR-148a increase upon B cell activation, maturation, and differentiation in germinal centers [192,193].

Experiments conducted by Pracht and colleagues demonstrated that mi-R148a plays an essential role in the establishment and maintenance of humoral immune responses, particularly for long-lived plasma cells (essential for long-lasting humoral protection) [193]. Moreover, miR-148a may be involved in the maturation of immunoglobulins A (IgA)-producing intestinal plasma cells, a class of antibodies belonging to mucosal immune defenses [181,193]. It would therefore be interesting to assess miR-148a levels in B cells from people receiving or having received modified mRNA lipid nanoparticles, such as those marketed by Pfizer or Moderna. Of note, it was demonstrated that miR-148a is induced by T-box transcription factor (also called T-box expressed in T cells, also known as T-bet) and Twist1, two Th1 cells transcription factors, and its inhibition by antagomirs leads to T-cell apoptosis [194].

Moreover, studies have shown that global miR-148a expression impacts neuropilin-1 (NRP-1) expression [195,196]. Today, it is well known that the S1 subunit can bind neuropilin-1, also called NRP-1, through the interaction between its C-terminal end and the b1 domain of this receptor [197,198]. Although the ways in which this receptor or coreceptor is used by the virus still need to be further investigated, neuropilin-1 plays a role in this infection.

As Gudowska-Sawczuk and Mroczko detailed, NRP-1 exerts a lot of biological functions, including cell proliferation, CNS and vascular physiology and development, coagulation, and immunity [199]. Many immune cells express NRP-1, from dendritic cells and macrophages to lymphocyte populations [200,201]. Also present on alveolar macrophages [202], it is highly likely that the virus infects these cells via NRP-1 and uses them to spread into the lungs, inducing respiratory distress in the process [203].

Indeed, it appears that the virus and its associated S protein have been detected in macrophages present in BAL samples of patients with severe COVID-19, probably leading to a loss of these macrophages and thus to an increased susceptibility to lung infections and the development of hyperinflammation [203,204].

NRP-1 is also involved in the establishment of immune synapses and in the homeostasis of the immune system [200]. The interaction between Spike and NRP-1 may therefore have direct consequences on immune cells and their functions. Finally, it seems that the Spike protein of the Omicron strains presents a higher binding affinity to NRP-1 [205]. So, does the global decrease of miR-148a enable the virus to infect an organism by upregulating NRP-1 expression and, better still, act on elements of the immune system?

Interpretations and discussions about the data exposed above

**Figure 1:**
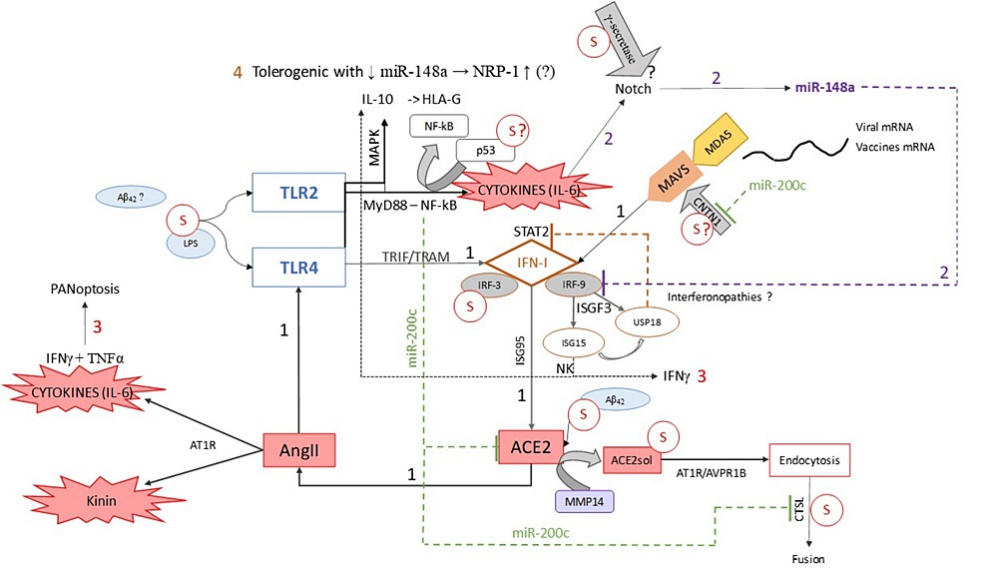
Summary diagram of all the data presented 1. TLR4/MDA5 - type 1 interferons (ISGs) - ACE2/Spike - Angiotensin II - TLR4 as amplification loop. 2. MiR-148a induction via Notch leading to a potential IFN-1 dysregulation. 3. ISG15 involvement in PANoptosis. 4. Development of tolerogenic activity with induction of HLA-G expression via IL-10 and a decreased miR-148a expression, which may promote NRP-1 expression. 5. The consequences of miR-200c expression are also shown. 6. Possible molecular interactions of the Spike protein are represented by question marks. Statement: Original illustration by Annelise Bocquet-Garçon, PhD

As can be appreciated, many scientific questions remain unanswered. Nevertheless, it is possible to schematize the various molecular interactions of the Spike protein with key elements of the innate immune system and to mark the main conundrums with question marks (Figure 1).

This diagram shows that the Spike protein can develop both tolerogenic and inflammatory activities.

In fact, it all depends on whether TLR4 and TLR2 are activated in the presence or absence of LPS. In the presence of LPS, TRIF/TRAM activation, combined with MDA5 activation, induces an IFN-I response and the amplification loop detailed above, i.e., "TLR4- IFN I (ISG)-ACE2/Spike-angensiotensin II-TLR4" loop. As a reminder, it has been shown that the Spike protein alone does not activate the non-canonical TLR4 pathway [132].

The type-I IFN response can be even more deleterious as ISG95 will induce ACE2 expression (while elevating NF-kB levels) [77], enabling the virus to infect cells that may not have originally been accessible to it, much like macrophages and peripheral blood immune cells [78]. The other potentially harmful effect of the type-I IFN response is the production of a truncated form of ACE2 (dACE2) via ISG15 [34,72]. Does this dACE2 bind to Spike protein, notably those of the new Omicron lineage variants, to induce endocytosis via ATIR or AVPR1B receptors [143-145]? It is a question that has intrigued scientists for some time [206]. Moreover, secreted ISG15 can induce IFN-γ by NK [101]. This production may be in line with that induced by the angiotensin II/AT1R axis overactivation. Thus, the type-I IFN response could then participate in PANoptosis, a phenomenon driven by the IFN-γ/TNF-α combination and resulting in potential lymphopenia [20,25,30].

Then, the inflammatory processes lead to an IL-6 production, activating Notch [177] and miR-148a synthesis [176]. miR-148a targets IRF-9 [164], decreasing its levels in microglial cells. This leads to a detrimental M1 phenotype for these macrophages, key players in the immune homeostasis of the CNS [165,175]. However, most importantly, an IRF-9 depletion can impair ISG15 and USP18 expression [99,166], two molecules involved in type-I IFN response control.

It is known that an overreactive response to interferons can lead to interferonopathies [169]. Interestingly, SARS-CoV-2 could join the "*Toxoplasma gondii*, other agents, *Rubella*, *Cytomegalovirus*, herpes simplex" (TORCH) pathogen group that have serious repercussions on fetuses and newborns, particularly in the CNS development, in which type-I IFNs may be involved [207,208].

Moreover, an IRF-9 disruption may lead to a p53 impairment [170] and an imbalance in the NF-kB/p53 relationship [209]. It was demonstrated that both NF-kB and p53 can induce the murine double minute-2 (MDM2) expression to inhibit each other, creating an equilibrium between their biological activities [210]. In addition, SIRT1 expression inhibits p53 and this gene is controlled by IRF-9 [170]. Thus, IRF-9 depletion can increase NF-kB activity, and a hyperinflammatory state can emerge via p53 blockade. In that context, the p53/Spike interaction must be investigated intensively [172]. If the impact of SARS-CoV-2 and its proteins on NF-KB/p53 crosstalk has already been fully reviewed by Milani's team [209], the role of the Spike protein in these mechanisms remains obscure. Of note, some compounds like polyphenols can upregulate p53 levels, displaying an anti-inflammatory activity in this way [209].

Interestingly, the miR-200c expression can be related to p53 activity [211], and thus, the analyses performed by Abdolahi and his colleagues [154] are intriguing. They demonstrated a significant decrease in miR-200c levels in COVID-19 patients hospitalized with an elevation of IL-6 amount, characteristic of an inflammatory state. These findings can indicate a p53 impairment but may also be due to other factors, such as the absence of associated Gram-negative bacterial infection or an infection with a less inflammatory SARS-CoV-2 variant, such as those of the Omicron lineage.

Furthermore, LPS alone can directly induce miR-200c expression, leading to a decrease in ACE2 expression, a phenomenon that can result in angiotensin II/AT1R overactivation and thus trigger a cytokine storm [153]. Thus, it is possible to suggest that the data obtained by the researchers studying this microRNA were in an associated bacterial coinfection, with a combination of Spike/LPS since antibiotics during a respiratory infection were removed from health protocols [149-152]. This hypothesis needs further investigations. Of note, miR-200c may also be involved in the mechanisms of type-I IFN hyperactivation since inhibition of CNTN1 results in the persistence of MAVS [155].

The so-called “vaccine” Spike protein can also trigger the proinflammatory amplification loop since it binds LPS more efficiently, notably due to the two proline modifications at positions 986 and 987 [84], and can interact with ACE2 [39,44]. This is reinforced by the fact that the modified mRNA of anti-COVID-19 injections, Pfizer or Moderna, is detected by MDA5 [34]. Inflammatory processes and the resulting cytokine production can lead to the development of CXCL10-related pathologies, such as those described in the body of the text, namely, multisystem inflammation syndrome (MIS) and myocarditis, as well as autoimmune pathologies, such as bullous pemphigoid [212].

It is well known that CXCL10 recruits neutrophils, eosinophils, and many other immune cells, such as lymphocytes, NK, monocytes, or mast cells, by binding to its receptor, CXCR3 [213-216]. In bullous pemphigoid, lymphocytes, mast cells, eosinophils, and neutrophils are involved, but the latter seem to play a predominant role [217]. Indeed, this pathology is initiated by an inflammatory cascade that leads to the production of autoantibodies directed against key components of hemidesmosomes, known as bullous pemphigoid antigens (BPAGs) with a 230-kD (BPAG1 or BP-230) and a 180-kD (BPAG2 or BP-180) protein, and the secretion of specific enzymes, MMP-9 and elastase, both being essentially produced by neutrophils. These two molecules destroy the extracellular matrix at the dermal-epidermal junction, leading to the formation of bullous vesicles on the skin surface [218,219]. Of course, cases of bullous pemphigoid have been reported in the literature after anti-COVID-19 injections [220-222].

At this stage, three remarks can be formulated.

First, targeting the cluster of differentiation 147 (CD147) can be of interest. Referring to the publication of Behl and his team, there are many arguments supporting and substantiating the hypothesis of CD147's involvement in COVID-19 pathology [223]. Also called basigin or extracellular matrix metalloproteinase inducer (EMMPRIN), CD147 is a highly glycosylated transmembrane protein involved in the recognition of molecules present in the same cells, notably in the same membrane (cis recognition), and those located extracellularly (trans recognition). In fact, this receptor has many ligands, such as cyclophilins, integrins, the γ-secretase complex, and other molecules. Moreover, it seems that galectin-3 binds to CD147 by recognizing a particular glycan motif, a repeating structure of galactose and N-acetylglucosamine called poly-N-acetylactosamine. This phenomenon induces the formation of CD147 clusters or promotes its interaction with β1-integrin, in both cis and trans manners, leading to the production of MMPs, including MMP9 and MMP14 [224-227]. Interestingly, the S1 spike subunit has a "galectin-3-like" sequence in its NTD, suggesting a direct interaction between Spike and CD147 [228]. Even if it was demonstrated by tests performed on cell cultures and on a mouse model (hCD147 mice) [229], the interaction between Spike and CD147 remains controversial. Indeed, one study tends to invalidate the hypothesis of a direct link between these two molecules [230]. However, as it was explained previously, the Spike/CD147 interaction may not be via RBD. Moreover, other studies suggest an indirect interaction via the nucleocapsid (N) of SARS-CoV and/or cyclophilin A (CyPA) [231-234]. 

Nevertheless, MMP9 can be involved during the SARS-CoV-2 infection, as other MMPs are - like MMP14 for the Omicron strains [143-145,235,236]. In vitro experiments carried out on specific cell cultures, especially on kidney cell line (A704), endometrium cell line (HEC50B), and ovarian cell line (OVTOKO) cultures, have demonstrated that metalloproteinases are involved in Spike-mediated fusion processes [237]. Finally, CD147 expression can be (also) induced via the angiotensin II/AT1R axis in the retinal pigment epithelium (RPE) cells [227,238].

Second, as previously reported, MMP9 can be secreted by neutrophils with other proteases, notably elastase. Interestingly, Veras et al. showed that the Spike protein of the virus, by interacting with its ACE2 receptor and being cleaved by TMPRSS2, can induce NETosis [239]. To explain it simply, this phenomenon is a form of neutrophil suicide, a death triggered by the release of a neutrophil extracellular trap (NET), i.e., a network of DNA, histones, antimicrobial peptides, and proteolytic enzymes, such as elastase and MMPs. Of course, this is a proinflammatory mechanism that can induce severe tissue damage [240]. NETosis has been identified in severe forms of COVID-19 [239,241] and is related to a T helper 17 (Th17) immune response [242]. However, the Spike protein degradation by neutrophil elastase leads to amyloid fibers [12]. The amyloidosis diseases are caused by abnormally folded proteins that form amyloid fibrils. These insoluble, non-degradable fibrils accumulate in various tissues and organs, sometimes leading to organ dysfunction or failure and death. Alzheimer's is the most characteristic and studied disease linked to these amyloid fibers. However, amyloidosis also affects the cardiovascular system and kidneys [243].

Two worthwhile publications report amyloidosis mechanisms either in connection with the virus or in connection with mRNA-modified injections: “COVID-19 Infection and Vaccination and Its Relation to Amyloidosis: What Do We Know Currently?” written by Wing Yin Leung et al. [244] and “The Potential Role of Ischaemia-Reperfusion Injury in Chronic, Relapsing Diseases Such As Rheumatoid Arthritis, Long COVID, and ME/CFS: Evidence, Mechanisms, and Therapeutic Implications” written by Douglas B. Kell and Etheresia Pretorius [245].

Third, the neuroinflammatory activities of the Spike protein, including the S1/Aβ42 complex, can be involved in both neurologic disorders and bullous pemphigoid. Indeed, it seems that people suffering from degenerative pathologies, such as Alzheimer's, are 10 times more prone to develop bullous pemphigoid because BPAG2, also called BP-180, is present both in basal keratinocytes and in the CNS, notably in the basal nucleus and hippocampus where Alzheimer's lesions have been observed. It is possible that CNS lesions or alterations expose the neuronal BP180 form, triggering an immune response, which, together with cross-reactions (BP-180 skin/BP-180 brain), initiates episodes of bullous pemphigoid [246]. Then, one may wonder to what extent the S1/Aβ42 complex contributes to the inflammatory mechanisms and what are the consequences. This is even more important as the Spike protein in Omicron variants appears to have enhanced amyloid properties [136].

In contrast to hyperinflammation, the Spike protein can orchestrate the complete shutdown of the immune response, via different processes.

The first one is the alteration of the type-I IFN response via its interaction with IRF-3 [115], but more likely, with CNTN1 [156]. Indeed, ISG15 prevents virus-dependent degradation of IRF-3 [166]. Thus, normally, the Spike/IRF-3 interaction does not really have a negative impact on IFN-I, at least in the context of mRNA-modified vaccines, since the papain-like protein of the virus dismisses ISG15 [95]. However, if Spike acts upstream, blocking MAVS signaling, for example, it could have a significant impact on the immune response initiation again in the context of modified mRNA vaccines. To our knowledge, only MDA5 can detect this modified mRNA, starting an innate immune response via MAVS [34]. However, here too, further investigations are required. 

In addition, the virus mutates its Spike protein to use elements of the IFN-I response - like IRF-3 to activate itself in the proteasome [115]. It can also hijack IFITMs to its advantage, as does the Spike protein of the alpha variant [127]. In any case, functional studies of Spike from emerging variants must be carried out as soon as possible, to anticipate its activity on type-I IFNs.

The second mechanism for blocking the immune response is to induce lymphopenia. In brief, the fusogenic capacities of the Spike protein and the formation of "cell-in-cell" structures can lead to lymphocyte depletion [121]. This is an important pathological mechanism because type-I IFNs seem powerless to stop it, as explained above. Although the Spike fusogenic capacities appear to be of lesser importance for the recent strains, they seem to be more sensitive to proteolytic degradation, with the establishment of new cleavage sites, including one for the neutrophil cathepsin G [148]. Other proteases are also involved in the Spike fusogenic activity, such as trypsin [14,247], plasmin [13], and proteinase 3 [148]. It is essential to investigate the properties conferred by these cleavages on the Spike protein, whether in terms of amyloid and inflammatory capacities or fusogenicity. 

Lymphopenia can also result from the direct infection of immune cells, including B and T lymphocytes by SARS-CoV-2 [20,54,248]. Here again, the mechanisms appear to be multiple but in a recent preprint, a team of scientists tried to demonstrate the infection of immune cells by SARS-CoV-2 (including B cells) via an antibody-dependent enhancement (ADE) phenomenon [54]. However, the ADE phenomenon alone cannot explain this facilitation of viral infection over a short period. Not only interactions between Spike and CD4 [191] but also upregulation of ACE2 or NRP-1 receptors may lead to increased SARS-CoV-2 infections. In this context, the results obtained by Abdolahi and his team are worrying, as nothing stops ACE2 expression [154]. In addition, reduced miR-148a expression can lead to NRP-1 overexpression [195,196]. These molecular mechanisms can pave the way for repeated viral infections.

Finally, the third process involves the tolerogenic activity of the Spike protein. According to the data presented at the end of the Results section, IL-10, rather than downregulation of miR-148a expression, can induce HLA-G expression [184]. Spike can induce IL-10 production, either through its proinflammatory capacities - a kind of attempt by the immune system to control inflammation - or through its interaction with CD4 and lymphocyte infection [191], or through the secretion of ISG15 [101], or through its activity on a certain population of immune cells, the myeloid-derived suppressor cells (MDSCs) or the granulocytic myeloid-derived suppressor cells (G-MDSCs). This class of immune cells can limit the clonal expansion of CD4+ and CD8+ T-cells by releasing substances, such as arginase-1, oxygen radicals, IL-10, transforming growth factor-beta (TGFβ), or prostaglandins. Together, these factors inhibit the proliferation of active lymphocytes but promote the expansion of regulatory T cells (Treg) [249,250]. Several factors are involved in the recruitment, differentiation, and proliferation of MDSCs, such as IL-6, IL-8, IFN-γ, and TNF-α [250,251], all cytokines detected either in SARS infection or following anti-COVID-19 injections and related to the Spike activity as broadly explained in this article.

Recently, a study in mice showed that repeated immunization with Spike leads to immunotolerance, with IL-10 production and an increase in Treg numbers [252]. Moreover, one of the clues that has paved the way for MDSC exploration is the level of IL-10 in samples from people suffering from a severe form of coronavirus. Indeed, the production of this cytokine is a predictive marker of the severity of the pathology. Moreover, neutrophils account for around 50% of cells in bronchoalveolar lavage samples from patients infected with SARS-CoV-2. Studies have demonstrated the expansion of neutrophil G-MDSCs in people severely affected by the virus; this proliferation has also been associated with lymphopenia [253].

Another indicator of this immunotolerance, which probably develops via IL-10, is the detection of IgG4 [254,255]. Due to their characteristics, IgG4 can dissociate into two half-molecules, i.e., one heavy chain and one light chain, and recombine with another IgG4 half-molecule. This mechanism is called “Fab arm exchange,” and, as a result, IgG4 acts monovalently, unable to form large immune complexes [256].

Furthermore, IgG4 antibodies cannot activate the classical complement pathway because of their low affinity for the complement component 1q (C1q), and they present a reduced binding affinity to the antibody-binding crystallizable fragment (Fc) gamma (γ) receptors (Fcγ receptors or FcγRs), except FcγRIIb, the only Fcγ receptor displaying an inhibitory function [257]. Of interest, IgG4 can be produced in repeated allergic exposure [257,258], and some anti-Spike IgE antibodies were characterized during a SARS-CoV-2 infection, especially in severe forms of COVID-19 [259]. Interestingly, this IgG4 production was detected following SARS-CoV-2 infection and after administration of anti-COVID-19 products, perfectly suggesting both the allergenic and tolerogenic side of the Spike protein and, thus, the production of this type of antibody by repeated exposure to this viral component [260-263].

Another point to mention is the direct inhibition of NK by the S1 subunit. Indeed, a study shows that S1 can decrease the expression of certain HLAs, which reduces the ability of cells to present viral antigens on MHC-I but increases the expression of HLA-E, which inactivates NK [264].

The lymphopenia and tolerogenic activities that Spike can induce on its own may explain its persistence in the body for months after infections or injections of mRNA-modified vaccines. Indeed, the Spike protein was detected in similar proportions in the blood of people who have received anti-COVID-19 injections and those who developed a long COVID syndrome after an infection [35,265,266]. Moreover, in Ogata et al.'s article [266], the Spike protein appears to persist in the bloodstream for up to three months, which is a relatively long period for an antigen. In addition, the so-called "vaccine" Spike protein, due to its two proline modifications, was detected in patients by mass spectrometry up to six months after administration of mRNA vaccines [267]. It is acknowledged that prolonged exposure to the same antigen can impair T cell functions. This was studied for chronic lymphocytic choriomeningitis virus (LCMV) and human immunodeficiency virus-1 (HIV-1), showing irreversible T cell exhaustion after prolonged exposure to antigens for two to four weeks [268-270]. CD8+ T cell exhaustion was observed after SARS-CoV-2 infections [271,272] and also after the first booster dose, i.e., a third injection of mRNA-1273 vaccine, alias Moderna [273].

Although these results are not yet peer-reviewed and the immune status of cancer patients is biased, repeated immunization experiments with the recombinant RBD domain of the Spike protein appear to show a decrease in antibody production and specific lymphocyte phenotypes characteristic of exhaustion, in a murin model [252]. It is possible to identify these lymphocyte phenotypes by techniques, such as flow cytometry and employing antibodies directed against certain membrane markers, inhibitors of cytotoxic function. T cells become anergic, unable to fight effectively against pathogens and their accompanying antigens [252,274,275].

## Conclusions

In this article, the dual character of the SARS-CoV-2 Spike protein, both inflammatory and tolerogenic, is clearly demonstrated with a hyperinflammatory trend in the presence of LPS. Immune tolerance may explain the persistence of Spike in the body for months, and repeated exposure to the same antigen, whether through infections or anti-COVID-19 injections, may lead to deleterious cumulative effects, via amyloid formations and neuroinflammatory activities. Overexpression of receptors, such as ACE2 due to IFN-I response or NRP-1 due to miR-148a downregulation, may create an action/reaction loop that enhances the infectious capacity of Omicron variants, explaining infectious breakthroughs in the days following anti-COVID-19 product administration.
